# Multilayer Nonwoven Inserts with Aerogel/PCMs for the Improvement of Thermophysiological Comfort in Protective Clothing against the Cold

**DOI:** 10.3390/ma15062307

**Published:** 2022-03-20

**Authors:** Agnieszka Greszta, Grażyna Bartkowiak, Anna Dąbrowska, Eulalia Gliścińska, Waldemar Machnowski, Paweł Kozikowski

**Affiliations:** 1 Department of Personal Protective Equipment, Central Institute for Labour Protection—National Research Institute, Wierzbowa 48 Str., 90-133 Lodz, Poland; grbar@ciop.lodz.pl (G.B.); andab@ciop.lodz.pl (A.D.); 2 Institute of Material Science of Textiles and Polymer Composites, Faculty of Material Technologies and Textile Design, Lodz University of Technology, Żeromskiego 116 Str., 90-924 Lodz, Poland; eulalia.klata@p.lodz.pl (E.G.); waldemar.machnowski@p.lodz.pl (W.M.); 3 Department of Chemical, Biological and Aerosol Hazards, Central Institute for Labour Protection—National Research Institute, Czerniakowska 16 Str., 00-701 Warsaw, Poland

**Keywords:** aerogel, PCM, protective clothing against cold, thermal regulating, thermophysiological comfort, DSC analysis

## Abstract

This study aimed to assess the developed nonwoven inserts with aerogel/PCM (phase change material) microcapsules for use in protective clothing against cold in terms of properties related to thermophysiological comfort. These inserts were obtained by the thermal bonding of a multilayer system consisting of needled-punched nonwovens and silica aerogel particles and/or PCM microcapsules evenly distributed between them. The influence of aerogel and PCM microcapsules on the basic physical properties of inserts, their microstructure, air permeability, and water vapor resistance was investigated and analyzed. The thermal insulation properties of inserts were assessed based on thermal conductivity results. The inserts with PCMs were also tested for their ability to regulate the temperature in the undergarment microclimate using the differential scanning calorimeter (DSC) and the “skin model” device. The research showed that the use of aerogel allowed for reducing the thermal conductivity of the insert by approximately 13% compared to the insert without additives. The high values obtained of the melting and crystallization enthalpy of inserts with PCMs confirmed their high efficiency in the heat absorption and release. Thus, the use of aerogel and PCMs in protective clothing against cold seems to be an effective solution for improving its protective properties and actively adapting its thermal insulation to the changing temperature conditions and the activity level of employees.

## 1. Introduction

It is extremely difficult to achieve full comfort in the typical protective clothing against the cold. To obtain an appropriate level of protection against the cold, such clothing is usually made of thick, heavy, multilayered systems of materials that restrict the freedom of movement of the wearer to a greater or lesser extent. In addition, during intense physical effort, the garment often becomes wet as a result of the release of significant amounts of sweat by the user, which reduces the thermal insulation of clothing and increases the feeling of discomfort [1,2]. This problem also appears in the work environment with high variability of temperature conditions.

To work in such conditions, clothing with regulated thermal insulation would be best. One of the solutions to improving comfort in protective clothing against the cold can be the use of electric heating inserts [1,2,3,4,5]. However, these elements only provide heat to the body areas above which they are installed in the garment, while other areas are prone to cooling down. A similar effect is achieved by using chemical heaters for clothing. The heat release occurs as a result of a chemical reaction that takes place between the chemical substances enclosed in the packages after their activation, e.g., by temporary kneading [3,6,7]. The lifetime of such inserts is limited. There are also examples of heated clothing using phase change materials (PCM) [8,9]. Such clothing generally provide a rather short heating duration because of the large mass of PCM required to achieve effective heating.

In order to improve comfort in protective clothing against cold and by reducing its weight, attempts were also made to apply shape memory alloys (SMA) to clothing [10,11,12]. It was assumed that the clothing would allow for active adjustment of its thermal insulation by changing the height of the SMA elements to the ambient temperature. However, this effect turned out to be too small; therefore, such clothing has not been introduced to the market thus far. The reasons for this are also the high costs of producing SMA elements as well as the time-consuming process of their application to clothing. Poikayil et al. proposed heating and cooling clothing with Peltier cells, intended for use in extreme weather conditions [13].

Modular clothing, proposed by Marszałek et al. [14], is a much simpler and cheaper solution to improve the thermophysiological comfort of employees in a cold environment. This garment allows for the easy adjustment of thermal insulation to the individual needs of the employee by selecting the appropriate clothing products from the 7-piece set. In clothing in areas requiring the greatest insulation, the authors proposed the use of a thicker nonwoven fabric. Studies with volunteers have shown that this garment is more effective in providing thermal comfort than previously used clothing. However, this type of solution requires putting on or taking off selected elements of the set (e.g., jacket, vest).

A completely new solution is the development of protective clothing against cold, which would be a combination of thin insulating material with the addition of ultralight aerogel particles and material with thermoregulatory properties containing phase change materials (PCMs).

Currently, silica aerogel has the greatest practical application. This aerogel, thanks to the developed internal structure with a huge number of pores, shows an exceptionally low density (not much high than air) and the lowest thermal conductivity of all known solids, i.e., ~0.02 W·m^−1^·K^−1^. This makes aerogels the best insulation materials in the world [15,16]. 

In a study carried out on aerogel materials for winter clothing, nonwovens were mainly used as a base for aerogel application [17]. Venkataraman et al. [18] proposed composites based on polyester/polypropylene nonwovens with silica aerogel particles, applied at the stage of thermal bonding of fibers in the nonwoven web. An interesting method of applying aerogel particles to the nonwovens was proposed by Xiong et al. [19] This method encapsulated aerogel granules inside a three-layer composite in small holes made in the middle layer (i.e., high-loft nonwoven). The upper and lower layers of the composite were the thin thermo-bonded nonwovens, which were joined to the middle layer in the lamination process. In turn, a team of scientists from the University of Leeds in Great Britain developed a special two-layer nonwoven fabric with channels filled with aerogel particles using Spunlace technology [20]. Studies have shown that after placing the material on an adjustable heated mat with a temperature of 33 °C (simulating the temperature of human skin), the temperature on the surface of aerogel channels was on average 2.5 °C lower than the air-filled channels. This proves that aerogel is more effective in inhibiting heat loss than air itself. Another, more and more frequently used method of producing aerogel nonwovens is the electrospinning method [21,22,23]. Venkataraman et al. [23] conducted the electrospinning process using a spinning solution obtained by dissolving polyvinylidene fluoride (PVDF) or polyurethane (PU) in dimethylformamide and adding silica aerogel in the form of granules (0.1–0.7 mm). The results confirmed that the aerogel effectively increased the thermal insulation properties of nanofiber materials. This type of nonwoven material has particularly great potential for use in protective clothing against the cold due to the fact that it is extremely thin and light, and therefore it can significantly reduce the weight and bulk of the garment.

In the case of aerogel materials, the dusting of aerogel is a common and troublesome problem. To solve this problem, the lamination process of materials (e.g., between the membrane layers) is sometimes used [24,25,26]. The composites of this type are commercially available on the market, among others under the Aerotherm^®^ [27], AG-T [28] or PimaLoft [29] brand. However, their serious disadvantage is their lack of breathability [30]. Therefore, research is still being conducted into the new methods of aerogel applications, which would allow to not only improve their thermal insulation properties but could also ensure physiological comfort.

Phase-change materials (PCMs) are chemical compounds that enable active temperature regulation in the undergarment microclimate, which is extremely important for ensuring comfort in clothing. These materials can absorb, store, and release large amounts of latent heat during the changing of their physical state (e.g., from solid to liquid and vice versa) [31,32]. The use of PCMs in the textile materials provides a pleasant cooling effect to the user of clothing, e.g., during increased physical effort, thanks to the melting of PCMs and absorption of the heat generated by the body and/or the feeling of heating effect due to the release of heat by PCMs during their crystallization [9,33]. Significant parameters of PCMs determining the efficiency of their operation are total enthalpy (latent heat) and thermal conductivity [31]. The value of the total enthalpy of PCMs (i.e., ΔH) indicates how much heat energy per unit mass can be absorbed or released by the PCMs during the phase-change process. A higher enthalpy gives a stronger and longer cooling or heating effect. In turn, the thermal conductivity of PCMs is responsible for the effective absorption or transfer of heat even at small temperature differences. For clothing applications, PCMs are usually used in the form of microcapsules, where the core is paraffin, and the shell is an organic polymer [34].

PCM microcapsules (MPCMs) can be applied to the textiles even at the stage of fiber production, e.g., during melt spinning [35] or electrospinning [36]. However, most studies concern the thermoregulatory materials with PCMs applied by coating [34,37], padding [38,39,40], or printing [38,41]. Research conducted by Sánchez et al. [34] showed that the heat capacity of fabrics coated with a polymer mixture with MPCMs increases with an increasing weight ratio of MPCMs to a binder, but only up to (30–35) wt.%. When more PCMs were used, the coating paste became thick and sticky, and the motorized film applicator produced irregular and heterogeneous films. As a result, the coating contained a lower amount of PCM microcapsules. The coating method was also used by Nejman and Cieślak [37]. Polyester woven and knitted fabrics were coated with polyacrylic pastes containing 40 wt.% MPCMs with a melting temperature of 18 °C and 28 °C, and their mixture was 50/50. The surface of the knitted fabric was evenly covered with MPCMs, and the exposed areas were visible on the woven fabric. Baltusnikaite et al. [42], to increase the comfort in military winter clothing, subjected lining fabrics to bath padding with the addition of two types of MPCMs with a melting point of approximately 34 °C and 27 °C. The use of MPCMs, which formed chemical bonds with the fibers, allowed an increase in the thermal resistance of the multilayer system of materials for military clothing by up to 20%. The melting enthalpy for the linings with these PCMs also reached quite high values (1.175–3.617) J·g^−1^. On the other hand, the SEM tests showed that MPCMs, which required the use of resin for bonding with the fabric, were damaged during coating, as a result of which the melting enthalpy was only (0.170–0.465) J·g^−1^. Bendkowska and Wrzosek [38] compared the thermoregulatory properties of needled and spunlaced nonwovens with MPCMs applied by padding and screen printing. In the case of padded nonwovens, the content of MPCMs ranged from 15 to 27 wt.%, while in the case of nonwovens with MPCMs applied by screen printing—from 20 to 32% wt.%. The DSC studies carried out by Nejman et al. [41] showed that the most effective in terms of heat absorption and release were knitted fabrics with MPCMs applied by screen printing, because they showed the highest values of the melting enthalpy (36.5 J·g^−1^) and crystallization (36.9 J·g^−1^). The effect was slightly lower for coated knitted fabrics, and it was weakest for padded knitted fabrics.

Knowing the benefits of the separate application of aerogels and PCMs in textiles, it is expected that their simultaneous use in protective clothing against the cold will allow for a synergistic thermoregulatory action. Application of just aerogels would only provide passive protection against the cold, and in the case of PCMs, the required basic thermal insulation level constant over time would not be achieved. Only the combination of a very low mass and low thermal conductivity of aerogels with the endothermic effect of PCMs in a specific temperature range and the direction of phase change will allow the intensification of the thermoregulatory effect under changing working conditions in a cold environment. Thus far, no research has been presented in the literature regarding the simultaneous use of aerogels and PCMs in protecting clothing against the cold. However, works have studied using materials for use in firefighting clothing. Shaid et al. [43] developed a special thermal layer for firefighter clothing where the outer side was coated with silica aerogel particles while the inner side was coated with aerogel/PCMs composite powder. This allowed for the extension of the time to reach the pain threshold. This type of material combination was also used by Zang et al. [44] Their study showed that the best thermal protection performance (TPP) could be obtained by using PCM microcapsules with a phase-change temperature of 45 °C. Shaid et al. [45] found that using a combination of aerogels and PCMs in the thermal layer allowed the ignition time to be extended to 5.5 s, while the average ignition time for the thermal layer with only PCMs was 3.3 s. Moreover, the combination of aerogels and PCMs increased the thermal resistance of the material compared to the material only containing PCMs.

This paper presents a new simple manufacturing method of multilayer nonwoven inserts with aerogel and/or PCMs microcapsules, intended for use in protective clothing against the cold to improve the thermophysiological comfort of users. These inserts were tested and compared, i.e., in terms of microstructure (the bonding layers, uniformity of the materials) and properties related to physiological comfort. To assess the effectiveness of the inserts in terms of providing protection against low temperatures, the thermal conductivity of the inserts was analyzed, which was calculated on the basis of the tests results of their thermal resistance and thickness. In addition, the inserts containing PCM microcapsules were also assessed for thermoregulatory properties by conducting tests using a differential scanning calorimeter (DSC analysis) and “skin model” device.

## 2. Materials

Polyester fibers (1.7 dtex/38 mm) and bicomponent (sheath-core) BICO fibers (5.4 dtex/52 mm) were used as the raw materials for the manufacturing of needle-punched nonwovens for inserts with aerogel/PCM microcapsules. Polyester fibers are commonly used for the production of nonwoven inserts for protective clothing against the cold, because they are characterized by good thermal insulation properties and a high resilience [46,47]. 

Silica aerogel in the form of granules, manufactured by Cabot Co., Alpharetta, GA, USA [48], was used to manufacture the thermal-insulating nonwoven inserts. The characteristics of the aerogels are given in Table 1. The lowest possible thermal conductivity was taken as the main criterion when selecting the aerogel to ensure the greatest thermal insulation of the garment. Other factors include the lowest possible density, the hydrophobic surface, and the possibility of use both in subzero temperatures (due to the conditions in which the clothing will be used) and in high temperatures (due to the method of aerogel application).

PCM microcapsules, obtained from Microtek Laboratories Inc., Moraine, OH, USA, were used to manufacture the nonwoven inserts with a thermoregulatory function. The specificity of the PCMs used are presented in Table 2 [49]. The selection was mainly based on the melting temperature close to the temperature in the undergarment microclimate, the highest latent heat (to ensure the long-lasting and effective cooling effect of the wearer’s skin), and the lowest possible density.

### Preparation of Nonwoven Inserts with Aerogel/PCMs

The nonwoven inserts with aerogel/PCMs were made based on 4 layers of needle-punched nonwovens (each with a mass per unit area of approx. 80 g·m^−2^) and aerogel granules and/or PCM microcapsules sandwiched between these layers.

A fleece with the transverse arrangement of fibers was made from a mixture of polyester fibers and BICO polyester fibers in the proportion 60:40. A laboratory roller carding machine (Befama Sp. z o.o, Bielsko-Biała, Poland) equipped with a horizontal paver (Asselin, Elbeuf, France) was used to make the fleece. The fleece produced was subjected to the needling process on a needle loom with the upper needle plate (Asselin, Elbeuf, France), using the needle number of 40/cm^2^ and a needle depth of 12 mm.

**Figure 1:**
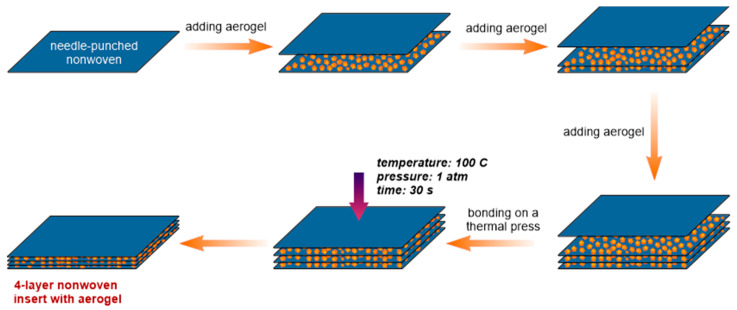
Manufacturing process of a multilayer nonwoven insert with silica aerogel granules.

Aerogel granules and/or PCM microcapsules were evenly distributed over a nonwoven sheet (30 cm × 30 cm). The sample was covered with a second nonwoven sheet. These activities were repeated until a system with 4 layers of nonwoven and 3 layers of additives (aerogel or PCM microcapsules) between them was obtained. This multilayer set of nonwovens and additives was thermally bonded on a thermal press for 30 s, using a temperature of 100 °C, and a pressure of 1 atm. At this temperature, the sheath of the BICO fibers with a lower melting point (than that of the core) melts, causing bonding points with nearby fibers, both BICO fibers and regular PES fibers. The reference sample (without additives) was produced in the same way, but by using a shorter pressing time (approximately 15 s) due to its smaller thickness and its higher thermal conductivity. A scheme of the manufacturing process of nonwoven inserts based on the addition of an aerogel is shown in Figure 1.

To assess the impact of various additives and their mass on the thermophysiological comfort properties of the inserts, two inserts with only aerogel granules, two inserts with PCM microcapsules, and one insert containing both aerogel and PCM microcapsules were developed. The 4-layer nonwoven insert without any additives was used as a control sample. The composition of the individual samples is given in Table 3. As a consequence of the implementation of additives to the insert structure, the thickness of these inserts increased. This is a result of the technological process and could not be avoided. Therefore, for the purpose of analysis of thermal performance of the developed samples, thermal conductivity was chosen that is independent of the sample’s thickness.

## 3. Methods

### 3.1. Determination of Thickness and Mass Per Unit Area

The thickness of the nonwoven inserts was measured in accordance with EN ISO 5084:1996 [50] using a thickness gauge RAINBOW T (Schröder Prüftechnik, Weinheim Germany) with a presser disc with the diameter of 50.5 mm and pressure of 1 kPa. 

The mass per unit area was tested according to EN 12127: 1997 [51] using 5 samples with dimensions of 100 mm × 100 mm. To determine their mass, an electronic laboratory balance PS 1200.R2 (Radwag, Radom, Poland) with an accuracy of 0.01 g was used.

### 3.2. Microstructure Analysis by SEM

The cross-sectional microstructure of the nonwoven inserts was analyzed from the micrographs and taken with a cold-field emission scanning electron microscope SU8010 (Hitachi, Tokyo, Japan) at an accelerating voltage of 5 kV. To minimize the sample damage during preparation of the cross-section, the samples were placed in a freezer for half an hour at −80 °C. This stiffened the structure of material and allowed for non-invasive cutting with a scalpel. This method of preparing samples of thermoplastic polymer materials for SEM analysis is used by many researchers [52,53]. Before starting the SEM analysis, the surface of sample was covered with a thin layer of gold using an ion-sputtering machine Q150T ES (Quorum Technologies, Lewes, UK). 

### 3.3. Thermal Conductivity Analysis

The thermal conductivity of nonwoven inserts was calculated from the tests results of their thermal resistance and thickness using Equation (1):

(1) $$\lambda =\frac{d}{100\cdot R_{ct}}\;\;\;\;\;\;\;\left[W\cdot m^{-1}\cdot K^{-1}\;\right]$$

where *d* is the thickness of insert (m); *R_ct_* is the thermal resistance of insert (m^2^·K·W^−1^).

The thermal resistance measurement was conducted according to EN ISO 11092: 2014 [54] on a “skin model” device (ATT Władysław Tarnowski Company, Poland), equipped with a thermally insulated measuring plate with dimensions of 200 × 200 mm with temperature control. A sample of the nonwoven insert was placed on a measuring plate and heated to a temperature of (35.0 ± 0.1) °C. The tests were carried out in a climatic chamber at a constant temperature (20.0 ± 0.1) °C, relative humidity (65 ± 3)% and air speed (1.0 ± 0.05) m·s^−1^. After reaching the steady-state condition, the thermal resistance of the nonwoven insert was calculated using Equation (2):

(2) $$R_{ct}=\frac{A\cdot \left(T_{m}-T_{a}\right)}{H-\Delta H_{c}}-R_{ct0}\;\;\;\;\;\;\;\left[m^{2}\cdot K\cdot {\;W}^{-1}\right]$$

where *A* is the surface area of the measuring plate (m^2^); *T_m_* is the temperature of the measuring plate (°C); *T_a_* is the air temperature in the chamber (°C); *H* is the heating power supplied to the measuring plate (W); Δ*Hc* is the correction factor of the heating power (W); and *R*_*ct*0_ is the thermal resistance of the plate alone (m^2^·K·W^−1^).

### 3.4. Thermoregulatory Properties of Nonwoven Inserts with PCMs

#### 3.4.1. Differential Scanning Calorimeter Test

Differential scanning calorimetry (DSC) was one of methods that were used to assess the thermoregulatory properties of the nonwoven inserts with PCMs. This method allows for the quantitative assessment of the thermal effects occurring when the tested material is heated or cooled. The measuring principle is the simultaneous heating and then cooling of a sample of the tested material and the reference sample (often an empty crucible) placed in one furnace on the sensitive thermocouples. During the test, the temperature difference between the samples is measured. This difference occurs when the test sample absorbs or gives off more heat than the reference sample. This occurs when the material undergoes a physical or chemical change that is accompanied by absorption or release of heat.

DSC analysis is a very important test method that allows us to understand the thermoregulatory effect of PCM materials. During the test, as a result of increasing the temperature in the furnace, an endothermic transformation (melting) occurs in the PCM sample, accompanied by heat absorption by the PCMs. The temperature of the tested sample with PCMs will be lower than the temperature of the reference sample (empty cup) until the PCMs are completely melted. Based on the temperature difference between the tested sample and reference sample, the heat flux supplied to the tested sample is determined. As the sample cools, the PCMs crystallize, resulting in the release of heat by the PCMs. The temperature of the tested sample is then higher than the temperature of the reference sample.

The same happens when using clothing with inserts containing PCMs. When the human body begins to generate more heat, for example as a result of exercise, the skin temperature begins to increase, which results in the activation of PCMs. Then the PCMs begin to melt and take away the heat emitted by the human body. As a result, the skin temperature is lowered to a comfortable level and is kept at this level until the PCMs are completely melted. After PCMs melt, the clothing with inserts (or the PCM inserts only) are cooled down below the crystallization temperature of PCMs. During the crystallization of PCMs, heat is released by PCMs. After crystallization, the PCM insert is ready for use again as a material regulating the temperature in the sub-clothing microclimate.

Thermal analysis of the nonwoven inserts with PCMs was performed using a DSC-6 heat flow scanning calorimeter (PerkinElmer Inc., Waltham, MA, USA). A sample of material (about 5 mg), after being closed in an aluminum cell, was placed inside the calorimeter and heated at a constant speed equal to 5 °C·min^−1^ from the temperature (−5 °C) to 50 °C. Then, the sample was cooled to (−5 °C) with the same speed. The tests were carried out under an atmosphere of dry nitrogen at a constant gas flow rate of 20 mL·min^−1^. The course of this study was recorded on a thermogram. For each variant of the nonwoven insert with PCMs and the reference insert, the enthalpy of melting and crystallization as well as the melting and crystallization temperature were determined.

#### 3.4.2. Testing the Cooling Efficiency of the Inserts with PCMs on a “Skin Model” Device

The “skin model” is a device commonly used to test the thermal resistance of materials. The control system used in the device is to maintain the temperature of the measuring plate at a constant level, i.e., 35 °C, by regulating the thermal power supplied to the plate.

To assess the effectiveness of inserts with PCMs as the cooling materials, the insert was placed on a measuring plate of “skin model” and the temperature of plate and heat loss flux (instantaneous heating power) were measured.

**Figure 2:**
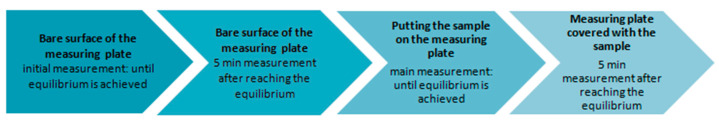
Scheme of testing the nonwoven inserts with PCMs on a “skin model” device.

The tests were carried out in isothermal conditions, i.e., on a heating plate with a constant temperature of 35 °C, in a climatic chamber at a temperature of 35 °C (i.e., corresponding to the temperature of the heating plate), a relative humidity of 65%, and an air speed of 1 m · s^−1^. The test procedure included 4 main stages listed in the diagram below (Figure 2). Under isothermal conditions, the heat exchange takes place only between the heating plate and the sample placed on the plate, but there is no heat exchange with the surroundings.

The samples were acclimatized for 24 h in a cooling chamber at 0 °C (i.e., at a temperature lower than the onset melting temperature of PCM microcapsules) before placing them on the surface of measuring plate. The tests were performed in 3 replications for each insert variant with PCMs and reference insert (variant PES).

Taking into account the recorded values of the heat loss flux in the “skin model” measuring plate (Q) and the surface area of this plate, the values of heat loss flux density (q) in watts per square meter (W·m^−2^) were calculated. The maximum heat loss flux density (q_max_) was determined for each variant of insert. This index allows for comparing the effectiveness of materials with PCMs regardless of the dimensions of heating plate of the “skin model” device.

### 3.5. Water Vapor Resistance and Moisture Permeability Index

Water vapor resistance tests were carried out in accordance with EN ISO 11092: 2014 [54] using the same apparatus as for the thermal resistance test, but on a metal porous sintered measuring plate with a system of water distribution channels. The surface of plate was covered with a cellophane foil. Both the plate temperature and the air temperature in the chamber were maintained at (35 ± 0.1) °C. The relative humidity in the chamber was set to (40 ± 3)% and the air flow velocity to (1.0 ± 0.05) m·s^−1^. After reaching the equilibrium conditions, the water vapor resistance of the sample was determined in accordance with Equation (3):

(3) $$R_{et}=\frac{A\cdot \left(\rho_{m}-\rho_{a}\right)}{H-\Delta H_{c}}-R_{et0}\;\;\;\;\;\;\;\left[m^{2}\cdot \mathrm{Pa}\cdot {\;W}^{-1}\right]$$

where *A* is the surface area of the measuring plate (m^2^); $\rho_{m}$ is the partial pressure of saturated water vapor on the surface of measuring plate at temperature *T_m_* (measuring plate temperature) (Pa); $\rho_{m}$ is the partial pressure of water vapor in the air at temperature *T_a_* (air temperature in the climatic chamber) (Pa); *H* is the heating power supplied to the measuring plate (W); Δ*H_c_* is the correction factor of the heating power (W); and *R*_*et*0_ is the water vapor resistance of the plate alone (m^2^·Pa·W^−1^).

Moreover, the moisture–permeability index *(i_mt_)* was also determined for each variant of insert. This index is generally used to determine the degree of evaporative cooling of material. It was calculated from the average thermal resistance and water vapor resistance of the insert by Equation (4).

(4) $$i_{mt}=\frac{R_{ct}}{R_{et}}\cdot K$$

where *K* is a constant equal to 60.6515 Pa · °C^−1^.

### 3.6. Testing the Air Permeability 

The air permeability of the nonwoven inserts was tested in accordance with EN ISO 9237: 1995 [55]. The air flow meter FX-3300 (Textest AG, Switzerland) was used for the tests. The sample of the insert was clamped in a circular holder with an opening with a measuring area of 20 cm^2^, and the amount of air passing perpendicularly through the sample was measured with a set pressure difference between both sides of the sample of 100 Pa. The average air permeability for each variant of insert was calculated based on 12 measurements.

## 4. Results and Discussion

### 4.1. Effect of Aerogel and PCM Microcapsules on Thickness and Surface Mass of Nonwoven Inserts

**Figure 3:**
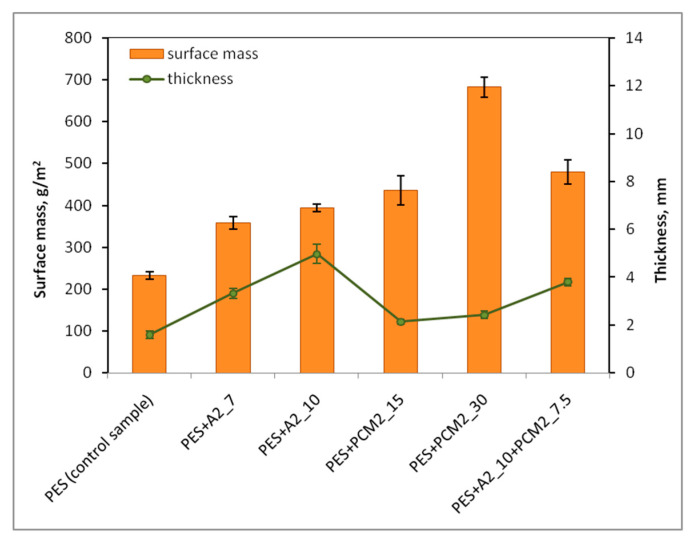
Thickness and surface mass of the nonwoven inserts.

The material thickness is one of the main parameters influencing the thermo-physiological comfort of the user of a piece of clothing. Increasing the thickness of the insulating layer is the most common way to increase the level of protection against cold in winter clothing. However, on the other hand, too large a thickness of these materials, especially those with a low water vapor permeability, may adversely affect the physiological comfort of the user. Likewise, the surface mass of the materials also should not be too high, so as not to create an additional load on the wearer and hence the clothing to become wet with intense exercise. Thus, the influence of additives (aerogel and PCM microcapsules) on the change of thickness and surface mass of the nonwoven inserts was analyzed. The test results of thickness and surface mass of inserts are shown in Figure 3.

The nonwoven insert without additives (PES) was characterized by a thickness of 1.59 ± 0.15 mm and a surface mass of 233 ± 9 g·m^−2^. The greatest increase in thickness in relation to the PES insert was recorded for the insert with the highest content of aerogel, i.e., PES + A2_10. The thickness of this insert was over three times greater and amounted to 4.97 ± 0.40 mm. This was due to the fact that the aerogel particles density is very low (Table 1), so the volume of 10 g of this additive was much larger than the volume of the same mass of PCM microcapsules. However, it should be noted that the thickness of this insert is smaller than the thickness of most insulation materials commonly used in protective clothing against the cold [56,57].

Comparing the PES + A2_7 and PES + A2_10 insert, characterized by different aerogel contents, it can be noticed that increasing the aerogel mass by only 3 g (for the surface area of 30 × 30 cm) resulted in an increase in the thickness of the insert by nearly 50%, with a slight increase in the surface mass by approximately 10% (from 358 to 394 g·m^−2^). This is due to the very low density of the aerogel used (120–150 kg·m^−3^) and its high porosity. Thanks to such properties of the aerogel, it is possible to significantly reduce the weight of protective clothing against the cold and thus reduce the physical load of the user.

The thickness of the insert containing 30 g of PCM microcapsules (PES + PCM2_30) was 2.42 ± 0.16 mm and was more than 50% less than the thickness of the insert containing 10 g of aerogel (PES + A2_10), but at the same time, this insert had a much greater surface mass (by 73%). Due to the high content of PCM, this insert can be very effective in terms of temperature control in the sub-clothing microclimate, but due to its weight it can be difficult to accept for users. 

Reducing the content of PCM microcapsules from 30 to 15 g made it possible to reduce the surface mass of the insert by 36% (from 682 to 436 g·m^−2^), while the thickness of the insert was only reduced by approximately 12%.

The result of thickness of the insert containing 10 g of aerogel and 7.5 g of PCM microcapsules (PES + A2_10 + PCM2_7.5), i.e., 3.80 ± 0.16 mm, was surprising. This insert was approximately 24% (i.e., 1.17 mm) thinner than the PES + A2_10 insert containing the same amount of aerogel but without the addition of PCM microcapsules. The lower thickness of this insert could have been due to the fact that the total mass of the 10 g aerogel was divided into two parts (5 g each) and not three (approximately 3.33 g each), as in the case of the PES + A2_10 variant. 

### 4.2. Microstructure of the Nonwoven Inserts with Aerogel and/or PCM Microcapsules

**Figure 4:**
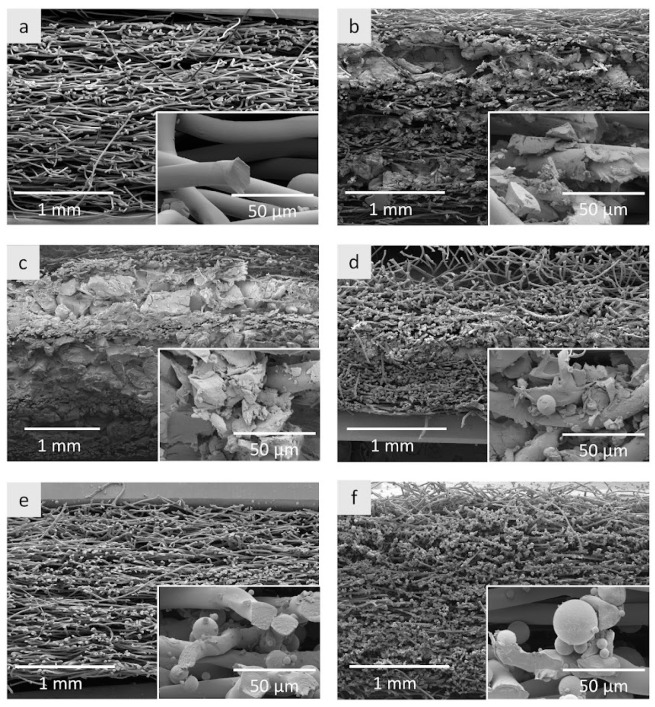
The SEM micrographs of nonwoven inserts: (**a**) PES, (**b**) PES + A2_7, (**c**) PES + A2_10, (**d**) PES + A2_10 + PCM2_7.5, (**e**) PES + PCM2_15, and (**f**) PES + PCM2_30.

Figure 4 shows the exemplary SEM micrographs of the developed nonwoven inserts (with and without additives) in a cross-section.

These images confirm that the layers of the inserts were successfully joined one to the other during the thermally bonded process on a thermal press, which obtained nonwoven multilayer products with a compact structure. In the structure of the insert without additives (Figure 4a), the layers of nonwovens are practically invisible. This is due to the use of thermoplastic fibers in nonwovens, which soften due to thermal treatment and allow adjacent layers to be bonded together.

The structure of this insert appears to be a bit looser and more porous than the inserts with additives, which is possibly related to the thermal processing time. The inserts with additives required a longer thermal treatment time so that the fibers in individual layers (especially in the middle layers) could soften and connect the layers.

In the case of inserts only containing aerogel (Figure 4b,c), a “sandwich” structure is clearly visible, i.e., alternating layers of nonwovens and layers formed by aerogel particles. The thickness of the aerogel layer is not fully uniform; in places, the aerogel particles are more visible, and in other places less due to the manual method of applying the aerogel.

Local changes in the thickness of this layer are particularly noticeable in the PES + A2_7 (Figure 4b) and PES + A2_10 + PCM2_7.5 (Figure 4d) inserts. To obtain a more even layer of aerogel, the method of its administration should be automated. As can be seen from the SEM images (Figure 4b–d), the aerogel particles are tightly packed in the inserts, but some of them, as a result of the applied pressure on the thermal press, cracked and crumbled, which is clearly visible at a magnification of 100. The problem of crushing aerogel particles is well known. This was pointed out by Kraner Zrim et al. [58], researching a laminated silica aerogel composite in terms of its suitability as thermal insulation in footwear protecting against the cold. The material was subjected to 30,000 bending cycles to simulate real conditions of use. In the SEM micrographs, the unbent composite had a homogeneous structure, while white areas appeared on its surface as a result of bending, indicating the presence of aerogel dust. It has therefore been found that the lamination of aerogel composites is an effective solution to prevent the spread of the aerogel to the environment. The problem with aerogel crushing was also noticed by Krzemińska et al. [59], who were coating fabric with a polymer dispersion with the addition of silica aerogel in the form of granules. Based on the SEM micrographs, the authors noted an approximate 50-fold reduction in the size of the aerogel particles as a result of applying a coating containing 6% aerogel to the fabric. The problem of crushing the aerogel was somewhat alleviated by adding a wetting agent to the polymer dispersion. Then, the size of the aerogel particles decreased 30 times.

The typical “sandwich” structure is not visible in the case of insert PES + A2_10 + PCM2_7.5 (Figure 4d). The aerogel is mainly visible in the middle layer, although it was also applied between the outermost layers of the inserts. On the other hand, PCM microcapsules are present in the entire volume, even though they were only applied between the last two layers of nonwoven fabrics from the bottom. Similarly, the SEM micrographs of nonwoven inserts with the addition of PCM microcapsules (Figure 4d–f) showed that the microcapsules are distributed throughout the entire volume of the inserts, even though they were applied in the same way as the aerogel particles, i.e., between the layers of nonwovens. This may be due to the size of the microcapsules, which are much smaller than the aerogel particles and, therefore, more easily migrate between the fibers. As it turns out, the nonwoven fabric layers are not a fully effective barrier for them because the microcapsules are visible even on the surface of inserts. PCM microcapsules partially adhere to the fibers (singly or more often in clusters) and partially fill the pores between the fibers. As in the case of aerogel inserts, there is also some unevenness in the distribution of these additives in the PCM inserts. The SEM micrographs (Figure 4d–f) show microcapsules that have broken, possibly due to the pressure applied during the sealing of the inserts. Increasing the number of microcapsules in the inserts from 15 g (Figure 4e) to 30 g (Figure 4f) caused a visible increase in the thickness of the cartridge, and at the same time, it was noticed that a larger number of microcapsules broke. This may be because, during thermal bonding, the microcapsules were protected to a lesser extent by the soft layers of nonwovens due to their large quantity and pressed against each other, thus breaking.

### 4.3. Effects of Aerogel and PCM Microcapsules on the Thermal Conductivity of Nonwoven Inserts

**Figure 5:**
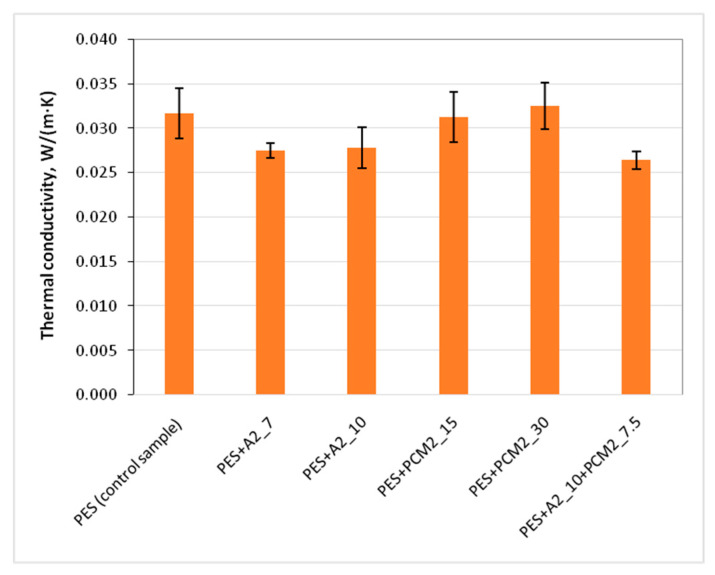
Thermal conductivity of the nonwoven inserts.

Thermal resistance is one of the main parameters informing about the thermal insulation properties of materials intended, e.g., for protective clothing against the cold. However, in order to evaluate the thermal performance of the developed nonwoven inserts characterised by varied thickness, thermal conductivity of the samples was calculated and analysed. The obtained test results of thermal conductivity of the developed nonwoven inserts are shown in Figure 5. The insert without additives (PES), together with the inserts with PCM addition, showed the highest thermal conductivity (above 0.030 W·m^−1^·K^−1^) among all the samples. It has occurred that addition of PCM microcapsules did not have a significant influence on the thermal conductivity of the inserts. 

In the case of the tested samples with aerogels, the effect of reduction of the thermal conductivity of about 13% was achieved. Aerogels are highly porous materials with a very low thermal conductivity, thanks to which they can significantly improve the thermal insulation properties of the materials to which they are applied, which is confirmed by numerous scientific works [19,20,60,61]. It was noticed that the increasing aerogel content in the sample did not have a significant influence on the thermal conductivity of the nonwoven inserts. 

In the case of the PES + A2_10 + PCM2_7.5 insert, which contained 10 g of aerogel and 7.5 g of PCM microcapsules, the lowest thermal conductivity 0 of 0.026 W·m^−1^·K^−1^ was achieved and was about 17% lower than in the case of insert without additives.

The obtained results should be assessed positively, as despite the introduction of additives to the structure of the inserts, in the case of none of the samples their thermal performance deteriorated. That could happen in the case of inserts with PCM microcapsules due to limitation of pore size between the fibers by the additives. However, results of the microstructure analysis (Figure 4) proved that PCM microcapsules partially adhere to the fibers and at the same time even a twofold increase of the thickness was observed in the case of inserts with PCM microcapsules (Figure 3). Those two factors could positively influence on keeping the pore size between the fibers and as a consequence, on not increasing the thermal conductivity of those samples. In the case of the inserts with aerogels, their very low thermal conductivity had a positive impact on the thermal performance of the nonwoven inserts, which was coherent with our expectations.

### 4.4. Effectiveness of Inserts with PCMs as the Thermoregulatory Materials

#### 4.4.1. DSC Analysis

**Figure 6:**
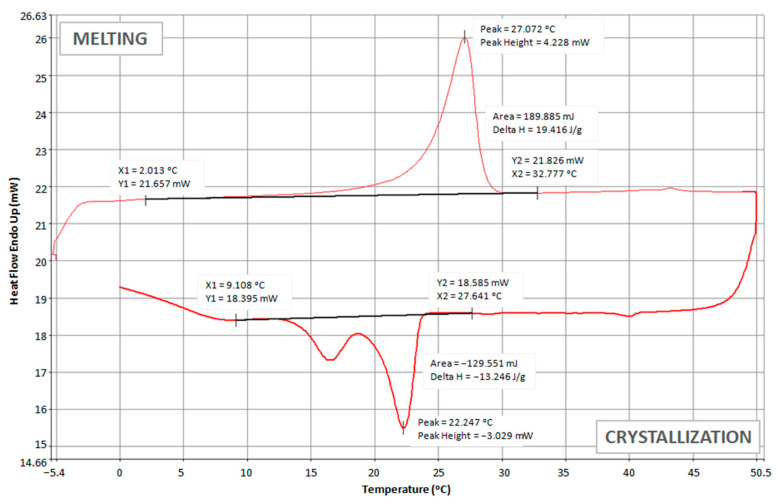
DCS thermogram of PES + A2_10 + PCM2_7.5 insert.

**Figure 7:**
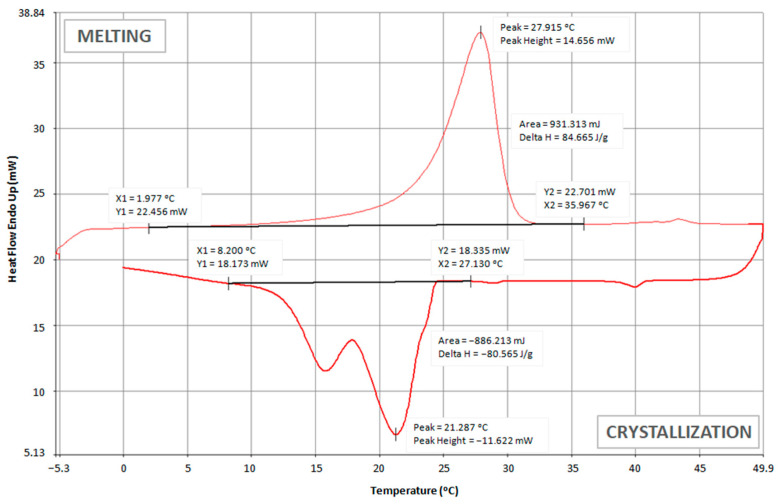
DCS thermogram of PES + PCM2_15 insert.

**Figure 8:**
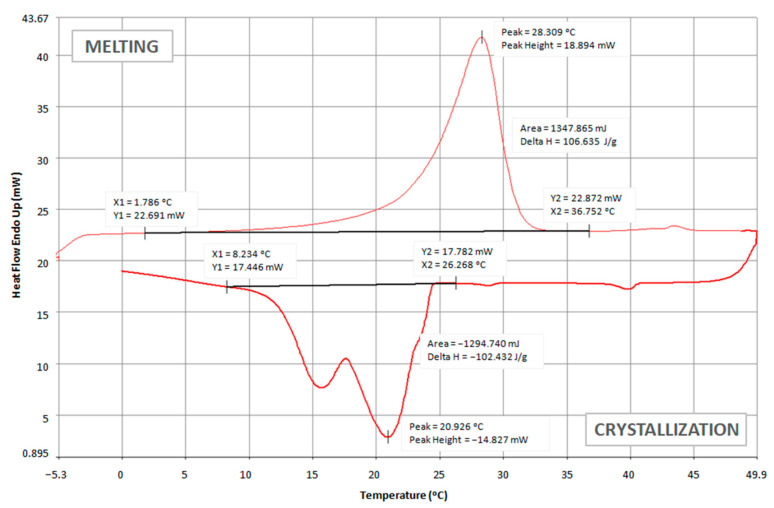
DCS thermogram of PES + PCM2_30 insert.

The thermoregulatory properties of nonwoven inserts with PCM microcapsules were tested, among others, by using a differential scanning calorimeter (DSC). The obtained test results are presented in the form of thermograms in Figure 6, Figure 7 and Figure 8 and Table 4.

Comparing the above thermograms of the developed nonwoven inserts with PCM microcapsules, a great similarity can be noticed, which results from the use of the same PCM microcapsules. For all the tested inserts, the peak melting temperature of PCMs was at a similar level of approximately (27.1–28.3) °C. These results are consistent with the melting temperature range stated by the manufacturer of PCM microcapsules, i.e., (28 ± 2) °C [49]. For the insert with the lowest PCM content, i.e., 7.5 g (PES + A2_10 + PCM2_7.5), the melting process started at approximately 2.0 °C and ended at 32.8 °C (Figure 6). It was noticed that as the content of PCM microcapsules increased, their melting ended at higher and higher temperatures, and for the insert with 30 g of PCMs, it was only at 36.8 °C (Figure 8). This is logical since the melting process of the PCMs took longer due to the greater amount of PCMs and thus the greater thickness of this layer. During the cooling process of the tested inserts, two crystallization peaks were recorded—the first at a temperature of approximately (20.9–22.3) °C, and the second at a temperature of (15.7–16.5) °C.

A higher value of the enthalpy of melting means greater efficiency of the nonwoven insert with PCM in terms of absorbing excess heat emitted by the human body and higher ability to regulate the temperature in the sub-clothing microclimate. This is directly related to the thermophysiological comfort in clothing. The obtained results of the enthalpy of phase transformations indicate that the best thermal effects are shown by the insert marked as PES + PCM2_30. Its melting enthalpy is over 100 J·g^−1^, which is only about 53% lower than in PCM microcapsules alone. Nevertheless, such an enthalpy is sufficient to ensure a high cooling efficiency of the organism. In the case of the PES + A2_10 + PCM2_7.5 insert, the melting enthalpy was only 19.4 J·g^−1^, which was more than a four-fold decrease compared to the PES + PCM2_15 insert with a two-fold higher content of PCM microcapsules. A similar enthalpy value to the PES + A2_10 + PCM2_7.5 insert was obtained by Bartkowiak and Dąbrowska for the three-layer knitted fabric containing 95% Smartcell™clima fibers with the addition of PCMs, i.e., 19.5 J·g^−1^ [62].

#### 4.4.2. Assessment of Cooling Efficiency of Inserts with PCM Microcapsules: Tests on a “Skin Model” Device

**Figure 9:**
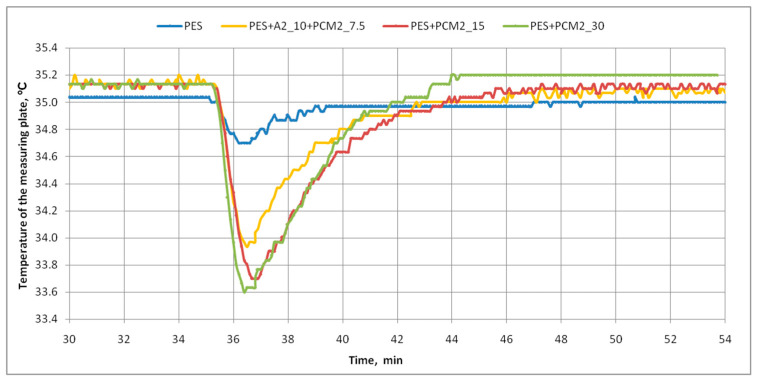
The course of changes in the mean values of the surface temperature of the measuring plate of the skin model (under isothermal conditions).

**Figure 10:**
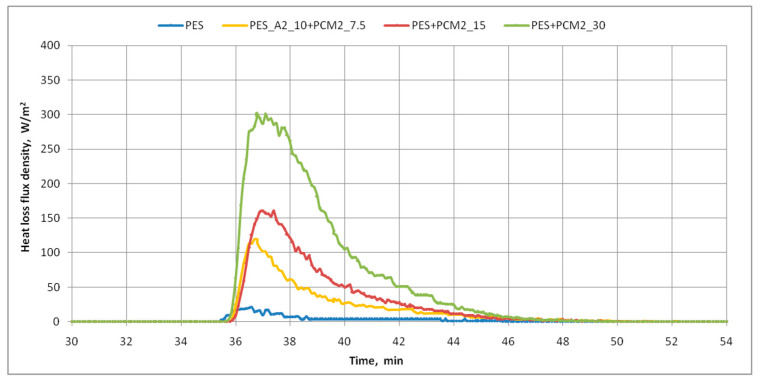
The course of changes in the mean values of heat loss flux density on the skin model (under isothermal conditions).

The research on the cooling efficiency of nonwoven inserts containing phase-change materials (PCMs) was also carried out using the skin model. The graphs below show the course of changes in the mean values of the surface temperature of the measuring plate of the skin model (Figure 9) and the density of heat loss flux (Figure 10) for three variants of inserts with PCM microcapsules and the reference insert. Based on the obtained graphs and additional data from the skin model, the maximum values of the heating power supplied to the measuring plate and the density of heat loss flux, as well as the minimum values of the temperature of the measuring plate, observed after the samples were placed on the skin model, were determined. These results are summarized in Table 5.

The above graphs prove that there are clear differences in the response of the thermal simulator, i.e., the skin model, depending on the tested inserted variant. In all cases, after the samples were applied, a significant decrease in the surface temperature of the measuring plate of the skin model was noted below the set value, i.e., 35 °C. It was found that the cooling efficiency of the measuring plate by the nonwoven inserts increased with increasing PCM microcapsule content in the insert. The insert with the highest PCM content, i.e., PES + PCM2_30, had the greatest impact on lowering the temperature of the measuring plate (Figure 10). It also resulted in the greatest increase in the heating power supplied to the skin model’s measuring plate, and thus the heat flux received by the insert (Table 5), because under isothermal conditions, both these quantities are the same. The increase in heating power resulted from the operation of the device’s control system, which constantly aims to maintain the temperature of the measuring plate at a constant level of 35 °C. In the case of the aforementioned PES + PCM2_30 insert, after its application, the deviation from the average surface temperature of the measuring plate before the application was the largest and amounted to 1.5 °C, while in the case of the PES reference insert, this difference was only 0.3 °C. This result indicates that the developed PCM nonwoven insert should effectively cool the body of the clothing user during temporary changes in ambient temperature or a short-term increase in physical activity. The use of a twice smaller mass of PCM microcapsules compared to the PES + PCM2_30 insert, i.e., 15 g, resulted in a similar reduction in the temperature of the measuring plate, i.e., by 1.4 °C, but at the same time, there was a 46% decrease in the maximum value of the heat flux density in relation to the insert with 30 g PCMs (from 300.8 to 160.6 W·m^−2^). In the case of the PES + A2_10 + PCM2_7.5 insert, the decrease in the surface temperature of the measuring plate in relation to its temperature before the placed of the insert was 1.2 °C, and the received heat flux density was 119.6 W·m^−2^, i.e., by approximately 26% lower than the insert with 15 g of PCM microcapsules.

### 4.5. Effect of Aerogel and PCM Microcapsules on Water Vapor Resistance of the Nonwoven Inserts

The water vapor resistance of fabrics is one of the main parameters influencing the physiological comfort of a garment user. The lower its value, the easier the fabric removes water vapor (sweat) from the skin’s surface and prevents it from condensing on the skin and on inner layers of clothing. The second important parameter of comfort textiles is the water vapor transmission index (*i_mt_*), which determines the degree of evaporative cooling of the material. This parameter indicates how quickly sweat will be evaporated by the material [63].

**Figure 11:**
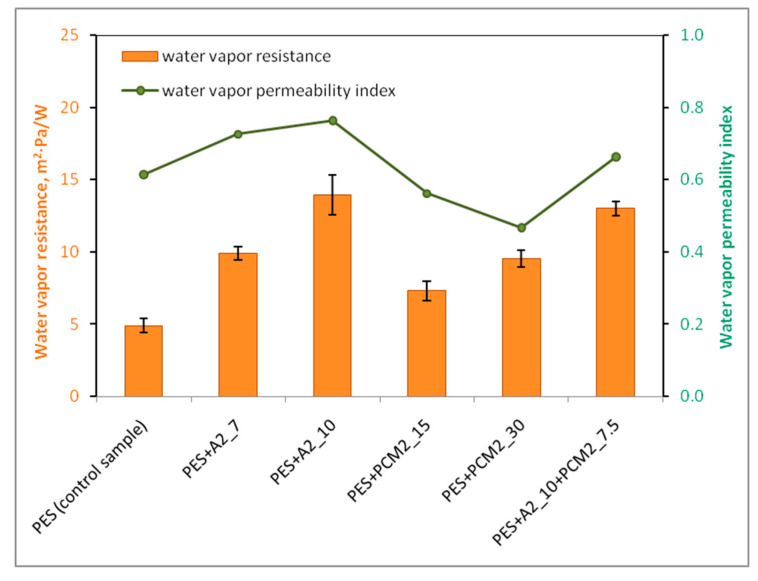
Water vapor resistance of the nonwoven inserts.

Among the tested samples, the most favorable, i.e., the lowest water vapor resistance, was demonstrated by the nonwoven insert without PES additives (4.90 m^2^·Pa·W^−1^) (Figure 11). According to the comfort rating system developed by the Hohenstein Institute (Table 6), [64,65], this insert is the only one that gives a water vapor resistance of no more than 6 m^2^·Pa·W^−1^), which proves its high efficiency in terms of sweat evacuation at a high activity rate of the user of clothing.

The remaining nonwoven inserts, due to the presence of additives (i.e., aerogel or PCM microcapsules) and the resulting greater thickness and lower porosity, were characterized by a significantly higher water vapor resistance. The porosity of textile material, consisting of the presence of pores in it (i.e., voids not filled with fibers or any additives), is one of the most important structural parameters of the material, which determines its ability to transmit water vapor [66]. It should be noted, however, that the water vapor resistance values obtained for these inserts did not exceed the upper limit of the range (6–13) m^2^·Pa·W^−1^, except for the insert with the highest content of aerogel (PES + A2_10). According to the classification given in Table 6, this means that the inserts were sufficiently permeable to water vapor to provide the user with comfort at a moderate activity rate.

The water vapor resistance of the nonwoven insert with 10 g of aerogel (PES + A2_10) was approximately 14 m^2^·Pa·W^−1^ and was nearly three times higher than the water vapor resistance of the reference insert (without additives). The main reason for this was the large thickness of the aerogel layers, which increased the thickness of the insert by more than three times and reduced its overall porosity. Despite the high water vapor resistance, the PES + A2_10 insert showed the highest value of the water vapor transmission index (*i_mt_*) of 0.77, which resulted from its exceptionally high thermal resistance (Figure 11). The high value of this index in the case of the aerogel insert indicates its better efficiency in terms of evaporative cooling.

Figure 11 clearly shows that all aerogel inserts have a better water vapor permeability index (0.67–0.77), not only compared to the reference PES insert (0.62), but also inserts only containing PCM microcapsules (0.47–0.57). Particularly noteworthy are the PES + PCM2_30 and PES + A2_7 inserts, which, despite a similar value of water vapor resistance (approximately 9.55–9.92 m^2^·Pa·W^−1^), drastically differ in terms of the *i_mt_* index. The PES + A2_7 aerogel insert, due to the higher value of this index, i.e., 0.73, will be able to more effectively manage the discharge of moisture through the clothing to the environment.

Research on the water vapor resistance of the aerogel nonwoven materials with a layered structure was also carried out by Bhuiyan et al. [61] These materials were developed for use in chemical- and heat-protective clothing. It was shown that with the highest aerogel content, i.e., 4 g (for the sample surface: 30 × 30 cm), the material showed a water vapor resistance of approximately 8.8 m^2^·Pa·W^−1^. This value was, by approximately 1.1 m^2^·Pa·W^−1^, lower than the water vapor resistance of the PES + A2_7 insert, which we developed, containing 7 g of aerogel. It should be noted, however, that the material made by Bhuiyan was about 2 mm thick, while the thickness of our PES + A2_7 insert was about 67% higher and amounted to 3.33 mm. It turned out that despite the lower thickness, Bhuiyan’s material was a slightly better insulator, as its thermal resistance was about 0.13 m^2^·K·W^−1^, while the thermal resistance of our insert was 0.12 m^2^·K·W^−1^.

### 4.6. Effect of Aerogel and PCM Microcapsules on Air Permeability of the Nonwoven Inserts

Air permeability, apart from water vapor resistance, is considered to be one of the basic parameters of textile materials that determine the comfort of wearing a garment. Thanks to it, it is possible to remove carbon dioxide from the sub-clothing space and to exchange heat with the environment, which is particularly important at a high activity rate. Air permeability strictly depends on the size and number of pores in the material. Materials with higher air permeability provide better thermophysiological comfort.

**Figure 12:**
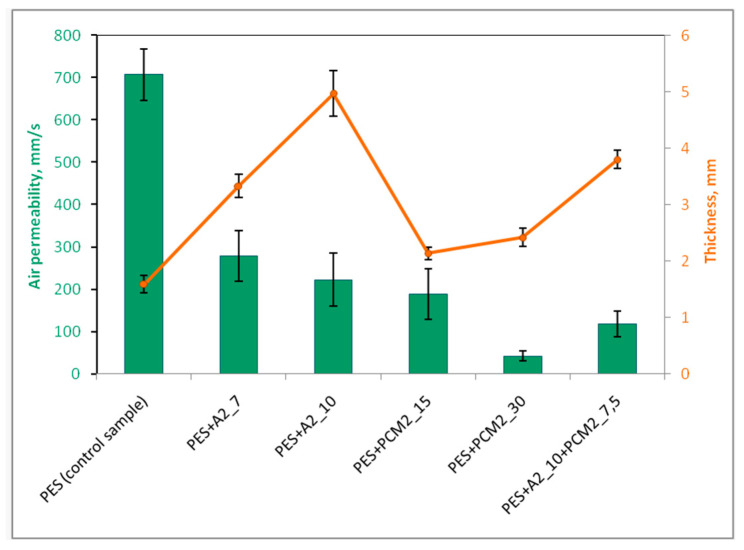
Air permeability of the nonwoven inserts.

Figure 12 clearly shows that the nonwoven insert without additives (PES) showed the highest air permeability, in the order of approximately 700 mm·s^−1^. The introduction of additives in the form of both aerogel and PCM microcapsules resulted in a significant reduction in this parameter, and a greater decrease was recorded in the case of PCM inserts. A similar tendency was noticed by Xiong et al. [67], examining spun-bonded polypropylene nonwovens with an electrospinning method applied with a PTFE layer with the addition of aerogel in the form of granules and/or PCMs. When using the same concentration of aerogel and PCMs (i.e., 3 g·L^−1^), the average air permeability of the material with aerogel was 134 mm·s^−1^, while the material with PCMs—121 mm·s^−1^, i.e., about 10% less. This may be because PCM microcapsules are inherently pore-free solids and therefore provide a certain barrier to air, especially at higher concentrations in the material. Moreover, it should be noted that the diameter of the PCM microcapsules was at least several times smaller than the size of the aerogel particles, which caused the PCMs to form a more compact, more densely packed layer and penetrate more easily into the pores of the nonwoven fabric, causing them to clog. Doubling the mass of PCM microcapsules in the insert (from 15 g to 30 g) was very unfavorable, with as much as a 4.5-fold reduction in air permeability. It was found that the variant of the PES + PCM2_30 insert with such a low air permeability (i.e., approximately 42 mm·s^−1^) would not be suitable for use in heat-protective clothing as a middle layer, as it would not be able to provide the wearer with a sufficiently high level of physiological comfort.

Inserts with aerogel also showed a decrease in air permeability with increasing aerogel concentrations, but this was not as abrupt as inserts with PCM microcapsules. Although aerogels are highly porous materials, their pore sizes (in the order of several to several dozen nanometers) are too small to ensure free air flow through the nonwoven insert. However, on the other hand, such a structure guarantees them very good thermal-insulation properties. The air permeability of the aerogel inserts was mainly determined by the arrangement of aerogel particles in the internal structure of the inserts. Due to the large size of the aerogel particles (approximately 100–700 µm) and their irregular shape, there were free spaces (pores) between these particles, which allowed for the air to flow through the inserts. Increasing the content of aerogel particles in the insert from 7 to 10 g resulted in their thickening, which closed some of the pores and reduced the air permeability of the insert by about 20% (from 279 to 222 mm·s^−1^). Similarly, Bhuiyan et al. [61], while conducting research on viscose–polyester nonwovens with an aerogel layer, also noticed that air permeability decreased with an increasing aerogel concentration. The difference between the material with the lowest and highest aerogel content, i.e., 1.0 and 4.0 g (for the surface area of 30 × 30 cm), was approximately 180 mm·s^−1^ (i.e., 56%). 

The values of air permeability of the PES + A2_7 and PES + A2_10 inserts significantly exceed the minimum value of air permeability (i.e., 100 mm · s^−1^) required by the EN 342 standard [68] for fabrics for clothing intended for work in a cold environment in rooms where the air velocity is lower. than 1 m · s^−1^. Therefore, it can be concluded that both these inserts are able to provide thermophysiological comfort to the clothing users under such conditions.

In this study, in the case of the insert containing both aerogel and PCMs (variant PES + A2_PCM2_7.5), the average air permeability was 118 mm·s^−1^ and was approximately 47% lower than the insert with aerogel alone (PES + A2_10). Nevertheless, this result is slightly better than the spun-bonded polypropylene nonwoven fabric developed by Xiong et al. [67] with a microporous PTFE layer with the addition of aerogel and PCMs. The air permeability of this nonwoven fabric with aerogel granules was approximately 82 mm·s^−1^, and with powder aerogel, it was approximately 61 mm·s^−1^.

Analyzing the relationship between the air permeability of the developed inserts and their thickness, it can be concluded that this factor affects the air permeability, but only in the case of inserts with the same type of additives. Increasing the thickness of aerogel insert by 48% (from 3.33 to 4.94 mm) resulted in a decrease in the air permeability by 20% (from 279 to 222 mm·s^−1^). A much greater effect of thickness on the air permeability was observed in the case of PCM inserts. Even a slight increase in the thickness of this type of insert of approximately 13% (from 2.14 to 2.42 mm) resulted in a 4.5-fold reduction in the air permeability (from 189 to 42 mm·s^−1^). Among the developed samples, the insert with the lowest thickness (1.59 mm), which is the reference sample, is characterized by the highest air permeability, i.e., over 700 mm·s^−1^.

## 5. Conclusions

A simple and effective method has been developed to manufacture thin insulating and thermoregulatory inserts containing aerogel and PCM microcapsules, which can significantly improve the ergonomics and comfort of using protective clothing against the cold. This method consists of making needle-punched PES nonwovens containing bicomponent fibers BICO, then creating a set from four layers of these nonwovens, between which additives, i.e., aerogel and/or PCM microcapsules, are introduced, followed by the thermal bonding of these layers.

The use of the developed aerogel inserts would eliminate thick nonwovens, commonly used in this type of clothing, and their replacement with a much thinner nonwoven fabric. The effect of aerogels on the improvement of thermal insulation properties of nonwoven inserts was confirmed by the results of laboratory tests. The research showed that the use of aerogel allowed for reducing the thermal conductivity of the insert by approximately 13% compared to the insert without additives.

The DSC thermal analysis of nonwoven inserts with the addition of PCM microcapsules showed their high efficiency in absorbing and releasing heat, thus regulating the temperature in the undergarment microclimate. The obtained values of the enthalpy of melting, which are a measure of the efficiency of the inserts in terms of cooling capacity, ranged from 19.4 kJ·g^−1^ for the insert containing 7.5 g of PCMs to over 106 kJ·g^1^ for the insert with 30 g of PCMs. Research on the use of the skin model in isothermal conditions also confirmed the highest cooling efficiency in the case of the insert with 30 g of PCMs. However, more comprehensive studies have shown that the more advantageous solution from the point of view of ensuring physiological comfort in protective clothing against cold is the use of an insert with half mass of PCM microcapsules (15 g). This insert is much lighter, has nearly 4.5 times higher air permeability, and approximately 23% lower water vapor resistance, and at the same time, provides a sufficiently high cooling efficiency, because its melting enthalpy is only approximately 20% lower than the 30 g PCM insert. Studies have shown that aerogel inserts, despite their greater thickness, allow for better air flow and constitute a smaller barrier against the penetration of water vapor than inserts with the addition of PCM microcapsules. However, it should be taken into account that the aerogel insert contained a lower mass of the modifier (additive), which could impact the obtained results. Further research on the inserts should be focused on assessing their durability after simulated conditions of use, e.g., washing cycles or repeated bending, taking into account their future use in protective clothing against cold.

## Figures and Tables

**Table 1:** Characteristics of silica aerogel [48].

Properties	Value
Composition	[(Trimethylsilyl)oxy]-modified silica
Form of aerogel	granules
Particle size	100–700 µm
Pore diameter	~20 nm
Particle density	120–150 kg·m^−3^
Surface chemistry	hydrophobic
Thermal conductivity	0.012 W·m^−1^·K^−1^ (at 25 °C)
Application temperature range	up to 300 °C

**Table 2:** Characteristics of PCM microcapsules [49].

Properties	Value
Composition	paraffin core/melamine shell
Form of microcapsules	powder
Particle size	15–30 µm
Particle density	~555 kg·m^−3^
Phase change	(28 ± 2) °C
Heat of fusion	≥155 J·g^−1^
Application temperature range	up to 250 °C

**Table 3:** Composition of the nonwoven inserts.

Variant	Mass of Additive, g		Thickness of Insert, mm	Scheme
	Aerogel Granules	PCM Microcapsules		
PES (control sample)	-	-	1.59 ± 0.15	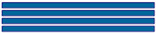
PES + A2_7	7	-	3.33 ± 0.21	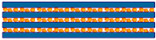
PES + A2_10	10	-	4.94 ± 0.40	
PES + PCM2_15	-	15	2.14 ± 0.11	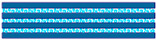
PES + PCM2_30	-	30	2.42 ± 0.16	
PES + A2_10 + PCM2_7.5	10	7.5	3.80 ± 0.16	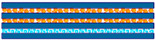

**Table 4:** Results of DSC analysis of PCM microcapsules and nonwoven inserts.

Sample	Melting		Crystallization	
	T_m_ [°C]	ΔH_m_ [J·g^−1^]	T_c_ [°C]	ΔH_c_ [J·g^−1^]
PCM2 microcapsules	27.0	226.9	22.3 (16.5) *	231.2
PES + A2_10 + PCM2_7.5	27.1	19.4	22.3 (16.5) *	13.3
PES + PCM2_15	27.9	84.7	21.3 (15.7) *	80.6
PES + PCM2_30	28.3	106.6	20.9 (15.7) *	102.4

* The value of the temperature of the second peak is given in brackets.

**Table 5:** Maximum values of heat loss flux (*Q_max_*) and heat loss flux density (*H_max_*) of the skin model and temperature values of the measuring plate of the skin model: average from 5 min before placing the sample (${\bar{T}}_{0}$) and minimum value after placing the sample (*T_min_*).

Variant	*Q_max_* (W)	*q_max_* (W·m^−2^)	${\bar{T}}_{0}$ ± SD (°C)	*T_min_* (°C)
PES	0.6	13,8	35.0 ± 0.0	34.7
PES + A2_10 + PCM2_7.5	4.8	119.6	35.1 ± 0.0	33.9
PES + PCM2_15	6.4	160.6	35.1 ± 0.0	33.7
PES + PCM2_30	12.0	300.8	35.1 ± 0.0	33.6

**Table 6:** Comfort rating system based on evaporative resistance of fabrics. Adapted from [64,65].

*R_et_* Value (m^2^·Pa·W^−1^)	Breathability	Wearer Comfort
0–6	Very good or extremely breathable	Comfortable at higher activity rate
6–13	Very good breathable	Comfortable at moderate activity rate
13–20	Acceptable breathability	Satisfactory, but uncomfortable at high activity rate
20–30	Slightly breathable	Moderate comfort at low activity rate
30+	Insufficient breathable	Uncomfortable and short tolerance time

## Data Availability

The data presented in this study are available on request from the corresponding author.

## References

[1] Mäkinen H., Jussila K., Wang F., Gao G. (2014). Cold-protective clothing: Types, design and standards. Protective Clothing: Managing Thermal Stress.

[2] Marszałek A., Bartkowiak G. (2013). Odzież ochronna do pracy w zimnym środowisku—Zasady projektowania i doboru. Bezpieczeństwo Pracy Nauka i praktyka.

[3] Song W., Lai D., Wang F. (2015). Evaluating the Cold Protective Performance (CPP) of an Electrically Heated Garment (EHG) and a Chemically Heated Garment (CHG) in Cold Environments. Fibers Polym..

[4] Park H., Hwang S.K., Lee J.Y., Fan J., Jeong Y. (2016). Impact of electrictrical heating on effective thermal insulation of a multi-layered winter clothing system for optimal heating efficiency. Int. J. Cloth. Sci. Technol..

[5] Lee H., Hong K., Lee Y., Kim S. (2017). User’s Voluntary Heating Behavior for the Programming of the Efficient Heating Mode of Smart Base Layer Clothing. J. Korean Soc. Cloth. Text..

[6] Cho H., Cho S. (2015). Optimal heating location for developing the heating smart clothing based on thermal response of body. Sci. Emot. Sensib..

[7] Irzmańska E., Kropidłowska P., Adamus-Włodarczyk A. (2021). Chemical Hand Warmers in Protective Gloves: Design and Usage. Autex Res. J..

[8] Gao C.H., Kuklane K., Holmér I. The heating effect of phase change material (pcm) vests on a thermal manikin in a subzero environment. Proceedings of the 7th International Thermal Manikin and Modelling Meeting.

[9] Babu V.R., Arunraj A. (2018). Thermo regulated clothing with phase change materials. J. Text. Eng. Fash. Technol..

[10] Yoo S., Yeo J., Hwang S., Hwang S., Kim Y.H., Hur S.G., Kim E. (2008). Application of a NiTi alloy two-way shape memory helical coil for a versatile insulating jacket. Mater. Sci. Eng. A.

[11] Nini M., Yehu L., Jiazhen H., Hongqin D. (2019). Application of shape memory materials in protective clothing: A review. J. Text. Inst..

[12] Lee J., Shin Y.W., Kim H.J., Baek B.K., Kim E. (2010). Prototype Intelligent Thermal Mountain Climbing Jacket Embedded with a Two Way Shape Memory Alloy. J. Korean Soc. Cloth. Text..

[13] Poikayil J.R., Francis J., Saju D., Suresh A., Varghese J. Peltier integrated heating & cooling jacket. Proceedings of the 2017 International Conference of Electronics, Communication and Aerospace Technology (ICECA).

[14] Marszałek A., Bartkowiak G., Dąbrowska A. (2018). Assessment of the effectiveness of modular clothing protecting against the cold based on physiological tests. Int. J. Occup. Saf. Ergon..

[15] Liao J., Gao P., Xu L., Feng J. (2018). A study of morphological properties of SiO_2_ aerogels obtained at different temperatures. J. Adv. Ceram..

[16] Anderson A.M., Carroll M.K., Aegerter M., Leventis N., Koebel M. (2011). Hydrophobic Silica Aerogels: Review of Synthesis, Properties and Applications. Aerogels Handbook. Advances in Sol-Gel Derived Materials and Technologies.

[17] Greszta A., Krzemińska S., Bartkowiak G., Dąbrowska A. (2021). Developing of high-insulating materials with aerogel for protective clothing applications—An overview. Int. J. Mater. Res..

[18] Venkataraman M., Militký J., Mishra R., Jandová S. (2018). Unconventional measurement methods and simulation of aerogel assisted thermoregulation. J. Mech. Eng..

[19] Xiong X., Yang T., Mishra R., Kanai H., Militky J. (2017). Thermal and compression characteristics of aerogel-encapsulated textiles. J. Ind. Text..

[20] Höffele S., Russell S.J., Brook D.B. (2005). Light-Weight Nonwoven Thermal Protection Fabrics containing Nanostructured Materials. Int. Nonwovens J..

[21] Shaid A., Wang L., Padhye R., Jadhav A. (2018). Needleless Electrospinning and Electrospraying of Mixture of Polymer and Aerogel Particles on Textile. Adv. Mater. Sci. Eng..

[22] Mazrouei-Sebdani Z., Khoddami A., Hadadzadeh H., Zarrebini M., Karimi A., Shams-Ghahfarokhi F. (2016). The effect of the nano-structured aerogel powder on the structural parameters, water repellency, and water vapor/air permeability of a fibrous polyester material. Mater. Chem. Phys..

[23] Venkataraman M., Mishra R., Militky J. (2018). Electrospun nanofibrous membranes embedded with aerogel for advanced thermal and transport properties. Polym. Adv. Technol..

[24] Jin L., Hong K., Yoon K. (2013). Effect of Aerogel on Thermal Protective Performance of Firefighter Clothing. J. Fiber Bioeng. Inform..

[25] Doshi D.A., Norwood C.M. (2014). Flexible Insulating Structures and Methods of Making and Using Same. U.S. Patent.

[26] Prevolnik V., Zrim P.K., Rijavec T. (2014). Textile technological properties of laminated silica aerogel blanket. Contemp. Mater..

[27] http://aerotherminsulation.com/about-aerotherm/technology.

[28] http://www.agel-tech.com/product.html?t=5.

[29] https://www.primaloft.com/news/primaloft-expands-use-of-its-high-performance-primaloft-cross-core-technology-with-increased-emphasis-on-sustainability/.

[30] Du Y., Kim H.E. (2019). A market research on the development trends of aerogel daily clothing. Fash. Text. Res. J..

[31] Mondal S. (2008). Phase change materials for smart textiles—An overview. Appl. Therm. Eng..

[32] Zwolińska M., Bogdan A. (2012). Związki zmiennofazowe w zastosowaniach techniczno-użytkowych i ergonomicznych. Bezpieczeństwo Pracy Nauka i praktyka.

[33] Hassabo A.G., Mohamed A. (2019). Enhancement of Thermo-Regulating Textile Materials Using Phase Change Material (PCM). Evol. Polym. Technol. J..

[34] Sanchez P., Sanchez-Fernandez M.V., Romero A., Rodriguez J.F., Sanchez-Silva L. (2010). Development of Thermo-regulating Textiles using Paraffin Wax Microcapsules. Thermochim. Acta.

[35] Sarier N., Onder E. (2012). Organic Phase Change Materials and Their Textile Applications: An Overview. Thermochim. Acta.

[36] Haghighat F., Hosseini Ravandi S.A., Nasr Esfahany M., Valipouri A. (2018). A comprehensive study on optimizing and thermoregulating properties of core–shell fibrous structures through coaxial electrospinning. J. Mater. Sci..

[37] Nejman A., Cieślak M. (2017). The impact of the heating/cooling rate on the thermoregulating properties of textile materials modified with PCM microcapsules. Appl. Therm. Eng..

[38] Bendkowska W., Wrzosek H. (2009). Experimental Study of the Thermoregulating Properties of Nonwovens Treated with Microencapsulated PCM. Fibres Text. East. Eur..

[39] Safavi A., Amani-Tehran M., Latifi M. (2014). Evaluation of dynamic thermal behavior of fibrous layers in presence of phase change material microcapsules. Thermochim. Acta.

[40] Karaszewska A., Kamińska I., Nejman A., Gajdzicki B., Fortuniak W., Chojnowski J., Slomkowski S., Sowinski P. (2019). Thermal-regulation of nonwoven fabrics by microcapsules of n-eicosane coated with a polysiloxane elastomer. Mater. Chem. Phys..

[41] Nejman A., Cieślak M., Gajdziski B., Goetzendorf-Grabowska B., Karaszewska A. (2014). Methods of PCM microcapsules application and the thermal properties of modified knitted fabric. Thermochim. Acta.

[42] Baltušnikaitė J., Valasevičiūtė L., Sankauskaitė A., Dubinskaitė K. (2015). Investigation of Thermo-regulating Properties of Multilayer Textile Package. Mater. Sci..

[43] Shaid A., Wang L., Padhye R. (2016). The thermal protection and comfort properties of aerogel and PCM-coated fabric for firefighter garment. J. Ind. Text..

[44] Zhang H., Song G., Su H., Ren H., Cao J. (2017). An exploration of enhancing thermal protective clothing performance by incorporating aerogel and phase change materials. Fire Mater..

[45] Shaid A., Wang L., Stanley M., Padhye R. (2018). Effect of Aerogel Incorporation in PCM-Containing Thermal Liner of Firefighting Garment. Cloth. Text. Res. J..

[46] Yang T., Xiong X., Petru M., Tan X., Kaneko H., Militký J., Sakuma A. (2020). Theoretical and Experimental Studies on Thermal Properties of Polyester Nonwoven Fibrous Material. Materials.

[47] Jussila K., Rissanen S., Aminoff A., Wahlström J., Vaktskjold A., Talykova L., Remes J., Mänttäri S., Rintamäki H. (2017). Thermal comfort sustained by cold protective clothing in Arctic open-pit mining—A thermal manikin and questionnaire study. Ind. Health.

[48] https://www.cabotcorp.com/solutions/products-plus/aerogel/particles.

[49] https://microteklabs.com/data-sheets.html.

[50] (1996). Textiles—Determination of Thickness of Textiles and Textile Products.

[51] (1998). Textiles—Fabrics—Determination of Mass per Unit Area Using Small Samples.

[52] Ma J., Deng H., Peijs T. (2007). Processing of Poly(propylene)/Carbon Nanotube Composites using supercritical CO_2_—Assisted Mixing. Eur. Polym. J..

[53] Amiri F., Moghadassi A., Bagheripour E., Parvizian F. (2017). Fabrication and Characterization of PES Based Nanofiltration Membrane Modifed by Zeolite Nanoparticles for Water Desalination. J. Membr. Sci. Res..

[54] (2014). Textiles—Physiological Effects—Measurement of Thermal and Water-Vapour Resistance under Steady-State Conditions (Sweating Guarded-Hotplate Test).

[55] (1995). Textiles—Determination of the Permeability of Fabrics to Air.

[56] Matusiak M., Kowalczyk S. (2014). Thermal-insulation properties of multilayer textile packages. Autex Res. J..

[57] Matusiak M., Szpak D. (2014). Wybrane metody oceny właściwości komfortu materiałów przeznaczonych na odzież zawodową stosowaną w środowisku zimnym—Część badawcza. Technol. Jakość Wyr..

[58] Kraner Zrim P., Mekjavic I.B., Rijavec T. (2016). Properties of laminated silica aerogel fibrous matting composites for footwear applications. Text. Res. J..

[59] Krzemińska S., Cieślak M., Kamińska I., Nejman A. (2020). Application of Silica Aerogel in Composites Protecting Against Thermal Radiation. Autex Res. J..

[60] Xiong X., Yang T., Mishra R., Militky J. (2016). Transport Properties of Aerogel-based Nanofibrous Nonwoven Fabrics. Fiber Polym..

[61] Bhuiyan M.A.R., Wang L., Shaid A., Jahan I., Shanks R.A. (2020). Silica aerogel-integrated nonwoven protective fabrics for chemical and thermal protection and thermophysiological wear comfort. J. Mater. Sci..

[62] Bartkowiak G., Dąbrowska A. (2012). Assessment of the Thermoregulation Properties of Textiles with Fibres Containing Phase Change Materials on the Basis of Laboratory Experiments. Fibres Text. East. Eur..

[63] Shaid A., Wang L., Padhye R., Bhuyian M.A. (2018). Aerogel nonwoven as reinforcement and batting material for firefighter’s protective clothing: A comparative study. J. Sol.-Gel. Sci. Technol..

[64] Reljić M., Stepanović J., Lazić B., Ćirković N., Cerović D. (2016). The change of water vapour resistance of materials used for the clothing production during exploitation. Adv. Technol..

[65] Houshyar S., Padhye R., Troynikov O., Nayak R., Ranjan S. (2015). Evaluation and improvement of thermo-physiological comfort properties of firefighters’ protective clothing containing super absorbent materials. J. Text. Inst..

[66] Matusiak M. (2013). Badania materiałów włókienniczych w zakresie ich zdolności do transportu wilgoci. Pomiary Autom. Kontrola.

[67] Xiong X., Venkataraman M., Yang T., Kucerova K., Militký J., Yang K., Zhu G., Yao J. (2020). Transport Properties of Electro-Sprayed Polytetrafluoroethylene Fibrous Layer Filled with Aerogels/Phase Change Materials. Nanomaterials.

[68] (2017). Protective Clothing—Ensembles and Germents for Protection against Cold.

